# Psychiatric comorbidities and all-cause mortality in epilepsy: A nationwide cohort study

**DOI:** 10.3389/fneur.2022.956053

**Published:** 2022-08-18

**Authors:** Kristijonas Puteikis, Edita Kazėnaitė, Rūta Mameniškienė

**Affiliations:** ^1^Faculty of Medicine, Vilnius University, Vilnius, Lithuania; ^2^Centre for Neurology, Vilnius University, Vilnius, Lithuania

**Keywords:** depression, comorbidities, premature death, seizures, suicide

## Abstract

**Background:**

People with epilepsy (PWE) having comorbid psychiatric conditions may be at greater risk of death. We aimed to determine the association between psychiatric disorders and all-cause mortality among PWE after adjustment for somatic comorbidities.

**Methods:**

Based on data from the National Health Insurance Fund, a Cox survival analysis was done within a retrospective open cohort of all PWE (≥12 years) in Lithuania between January 2014 and June 2020. Cox models comparing mortality between PWE with or without psychiatric comorbidities were adjusted for sex, age, hospitalizations, and the epilepsy-specific comorbidity index.

**Results:**

Of 47,964 PWE (age Md = 49, IQR = 34–62 years, 60.3% male, follow-up Md = 4.4, IQR = 2.1–6.1 years), 10,290 (21.5%) died during the study. The diagnosis of any psychiatric disorder (*n* = 26,137, 54.5%) was associated with increased mortality when adjusted for only sex and age (HR = 1.13, 95% CI = 1.09 to 1.18). After including the epilepsy-specific comorbidity index, the number of hospitalizations and hospital days in the analysis, only self-harm (HR = 1.55, 95% CI = 1.40 to 1.71) and substance use disorders (HR = 1.39 95% CI = 1.32 to 1.47), but not any psychiatric comorbidities (HR = 0.92 95% CI = 0.88 to 0.96) were related to elevated all-cause mortality. Mood, anxiety and behavioral disorders were associated with lower odds of mortality; however, they were rarely documented.

**Conclusions:**

Our results suggest that psychiatric comorbidities increase all-cause mortality among PWE through their association with coexisting somatic conditions as only substance use disorders and self-harm were independently related to elevated all-cause mortality. Future clinical interview-based studies should explore the relationship between mortality in epilepsy and psychiatric comorbidities while adjusting for somatic comorbidities.

## Introduction

People with epilepsy (PWE) are known to be at greater risk of premature mortality if compared with the general population (1). Seizures can lead to trauma, drowning or vehicle accidents, or directly cause sudden unexpected deaths in epilepsy (SUDEP) (2). SUDEP has long been regarded as one the most significant determinants of excessive deaths among PWE (3). However, recent findings suggest that “unnatural” causes of premature mortality, such as trauma, homicide, suicide, iatrogenesis, are equally important contributors to early deaths in epilepsy (4). Such causes of death are often directly associated with socioeconomic, cognitive and psychological issues coexisting with epilepsy (5). Especially important are psychiatric disorders, of which almost all are more prevalent among PWE than in individuals without epilepsy (4, 6). Psychiatric comorbidities in epilepsy have been associated with poor quality of life, worse response to antiseizure drugs or epilepsy surgery as well as higher healthcare costs (7, 8). However, the relationship between psychiatric disorders and death in epilepsy has only rarely been investigated (9–11). While it has already been shown that psychiatric comorbidities in epilepsy increase the odds of dying from external causes, their association with all-cause mortality is less pronounced (10). Further, if only specific psychiatric conditions are considered, they apparently do not increase mortality among PWE (9, 11). However, it remains unknown how the mortality of PWE with psychiatric disorders depends on the presence of other somatic comorbidities, which independently lower the odds of survival (11). In the current study, we test the hypothesis that psychiatric disorders among PWE are not associated with increased odds of all-cause mortality after adjustment for somatic comorbidities.

## Methods

The study is reported according to The REporting of studies Conducted using Observational Routinely-collected health Data (RECORD) guidelines (12).

### Study design and participants

We performed a retrospective survival analysis of a nationwide open epilepsy cohort based on data from the National Compulsory Health Insurance Fund (NCHIF) of Lithuania. The NCHIF reimburses local healthcare institutions in Lithuania for all eligible services after they transfer information to regional state health insurance offices that verify data and approve reimbursement claims (13). The database of the NCHIF contains person-level information, including demographic data, medical diagnoses, hospital admissions, and information whether a person is still alive. The country relies on compulsory health insurance, thus, data of up to 98% of inpatient and 90% of outpatient visits are processed through the NCHIF.

We extracted data of all individuals with epilepsy aged 12 or older between 1 January 2014 and 1 June 2020 (code G40 [epilepsy] with or without G41 [status epilepticus], according to the International Statistical Classification of Diseases and Related Health Problems, Tenth Revision, Australian Modification, ICD-10-AM). The age cut-off was selected for inclusion of adolescents with epilepsy but exclusion of children, which represent a group with more severe cases of refractory epilepsy. In Lithuania, the diagnosis of epilepsy is entered in electronic health records each time PWE use healthcare services reimbursable by the NCHIF (e.g., acquire a drug prescription for no longer than 6 months, visit outpatient clinics or are hospitalized). A previous study using the NCHIF database and having a similar case definition reported a prevalence of epilepsy of 11.9–12.7 per 1000 individuals (years 2016–2019) (14). While we were unable to validate the current database because of the anonymity of the study, a recent meta-analysis has shown that administrative healthcare databases can be used with confidence for the identification of PWE (15).

The dataset contained the sex and age of PWE at the start of the study, the number of hospitalizations and hospital days during the study period as well as a list of each person's comorbidities. Relevant somatic comorbidities were quantified by calculating the epilepsy-specific comorbidity index, which was developed and validated as a risk adjustment tool for population-based studies of PWE (11). Subgroups within the PWE sample were investigated based on psychiatric comorbidities or self-harm as follows: those having any psychiatric condition (ICD-10-AM codes F00–F99), mental disorders due to known physiological conditions (F00–F09), a substance use disorder (F10–19), schizophrenia or related disorders (F20–F29), mood disorders (F30–F39), anxiety and related disorders (F40–F48), behavioral syndromes (F50–F59), intellectual disabilities (F70–F79), or those identified with self-harm (X60–X84). Disorders of adult personality and behavior (F60–F69) as well as developmental (F80–F89), early-onset (F90–98), and unspecified (F99) disorders were excluded from the analysis because of small prevalence (<2%).

### Statistical analysis

The endpoint of the study was all-cause mortality before the end of the study period. First, a sex and age-adjusted multivariable Cox proportional hazards model was created to compare mortality between PWE with and without psychiatric comorbidities. Time was measured in days between the first diagnosis of epilepsy and the endpoint. Second, hospital-related variables (times hospitalized and hospital days) and the epilepsy-specific comorbidity index were included in the Cox regression as additional covariates. Finally, sensitivity analysis was done: the association of psychiatric comorbidities and mortality was retested after additional adjustment of the Cox model for the time of entry in the study, place of residence the type of epilepsy, the number of different diagnoses of epilepsy and after excluding individuals of <18 years. The type of epilepsy was defined as focal (ICD-10 codes G40.0, G40.1, G40.2), generalized (G40.3), encephalopathy and mixed (G40.4), special syndromes (G40.5), other epilepsy (G40.8), and unspecified epilepsy (G40.6, G40.7, G40.9), according to suggestions by Christensen and Sidenius (16).

The threshold for significance was set at *p* < 0.05 and all analyses were performed in SPSS v26.

## Results

After excluding 655 (1.3%) individuals with only status epilepticus and 45 (0.1%) individuals with a negative value of time-to-event, we identified 47,964 PWE to be included in the analysis (follow-up Md = 4.4, IQR = 2.1–6.1 years). Most individuals (29,917, 62.4%) had a diagnosis of focal epilepsy, 7,462 (15.6%)—of generalized epilepsy, 6,618 (13.8%)—of a special epileptic syndrome, 1,311 (2.7%)—of epileptic encephalopathy and mixed seizure types. There were 4,676 (9.7%) PWE diagnosed with “other” and 8,601 (17.9%)—with unspecified epilepsy. Within the sample, 38,795 (80.9%) PWE were diagnosed with a single type of epilepsy, 7,837 (16.3%)—with two, and 1,332 (2.8%)—with three or more types of the disease. The characteristics of all PWE as well as their subgroups based on psychiatric comorbidities are presented in Table 1. Unadjusted survival curves comparing PWE with and without specific psychiatric comorbidities are presented in Supplementary Figure 1.

**Table 1:** The characteristics of study participants.

**Comorbidity status**	**All PWE**	**Any psychiatric disorder**	**Mental disorders due to known physiological conditions**	**Substance use disorders**	**Schizophrenia and associated disorders**	**Mood disorders**	**Anxiety and related disorders**	**Behavioral syndromes**	**Intellectual disabilities**	**Self-harm**
Number of PWE (*n*, %)	47,964 (100)	26,137 (54.5)	13,486 (28.1)	9,136 (19.0)	1,368 (2.9)	3,689 (7.7)	5,574 (11.6)	1,791 (3.7)	2,680 (5.6)	1,999 (4.2)
Male/female (*n*, %)	28,912 (60.3)/ 19,052 (39.7)	15,909 (60.9)/ 10,228 (39.1)	5,489 (40.7)/ 7,997 (59.3)	7,530 (82.4)/ 1,606 (17.6)	813 (59.4)/ 555 (40.6)	1,379 (37.4)/ 2,310 (62.6)	2,520 (45.2)/ 3,054 (54.8)	926 (51.7)/865 (48.3)	1,542 (57.5)/ 1,138 (42.5)	1,501 (75.1)/ 498 (24.9)
**Characteristics**										
Age (median, IQR)	49 (34–62)	49 (35–62)	57 (44–70)	44 (35–53)	44 (30.25–55)	51 (38–62)	43 (29–57)	56 (42–69)	24 (17–36)	41 (31–51)
Hospitalizations (median, IQR)	2 (1–4)	2 (1–5)	3 (1–5)	3 (1–5)	3 (1–7)	3 (1–6)	3 (1–5)	3 (1–5)	1 (0–4)	4 (2–7)
Hospital days (median, IQR)	10 (1–29)	16 (4–40)	23 (7–51)	19 (7–43)	43 (11–100.75)	25 (7–59)	15 (3–41)	19 (5–44)	6 (0–25)	25 (10–57)
Epilepsy-specific comorbidity index (median, IQR)	1 (0–4)	2 (0–5)	3 (1–6)	1 (0–3)	1 (0–3)	2 (1–4)	1 (0–3)	3 (1–5)	0 (0–1)	1 (0–2)
**Outcome (** ***n*** **, %)**										
Died during the study	10,290 (21.5)	6,054 (23.2)	4,305 (31.9)	1,877 (20.5)	249 (18.2)	568 (15.4)	564 (10.1)	352 (19.7)	213 (7.9)	430 (21.5)
Emigrated during the study	593 (1.2)	224 (0.9)	34 (0.3)	115 (1.3)	8 (0.6)	26 (0.7)	81 (1.5)	7 (0.4)	13 (0.5)	30 (1.5)
Alive until end of study	37,081 (77.3)	19,859 (76.0)	9,147 (67.8)	7,144 (78.2)	1,111 (81.2)	3,095 (83.9)	4,929 (88.4)	1,432 (80.0)	2,454 (91.6)	1,539 (77.0)

*IQR, interquartile range*.

Being diagnosed with any psychiatric condition was related to elevated mortality after adjusting for sex and age (HR = 1.13, 95% CI = 1.09 to 1.18). In respectively adjusted Cox proportional hazards models, mood disorders (HR = 0.73, 95% CI = 0.67 to 0.80), anxiety and related disorders (HR = 0.53, 95% CI = 0.49 to 0.58) as well as behavioral syndromes (HR = 0.66, 95% CI = 0.60 to 0.74) were associated with a lower, while mental disorders due to known physiological conditions (HR = 1.20, 95% CI = 1.15 to 1.25), substance use disorders (HR = 1.36, 95% CI = 1.29 to 1.43), and self-harm (HR = 1.47, 95% CI = 1.33 to 1.62)—with a higher rate of mortality. After the addition of codes F06.3 (“Mood disorder due to known physiological condition”) and F06.4 (“Anxiety disorder due to known physiological condition”) alongside initial codes for mood (F30–39) and anxiety (F40–48) disorders, these conditions remained associated with decreased odds of mortality (HR = 0.80, 95% CI = 0.75 to 0.85 and HR = 0.56, 95% CI = 0.51 to 0.60, accordingly). There was no relationship between mortality and intellectual disabilities (HR = 0.98, 95% CI = 0.85 to 1.13) or schizophrenia and related disorders (HR = 1.13, 95% CI = 1.00 to 1.29).

**Figure 1:**
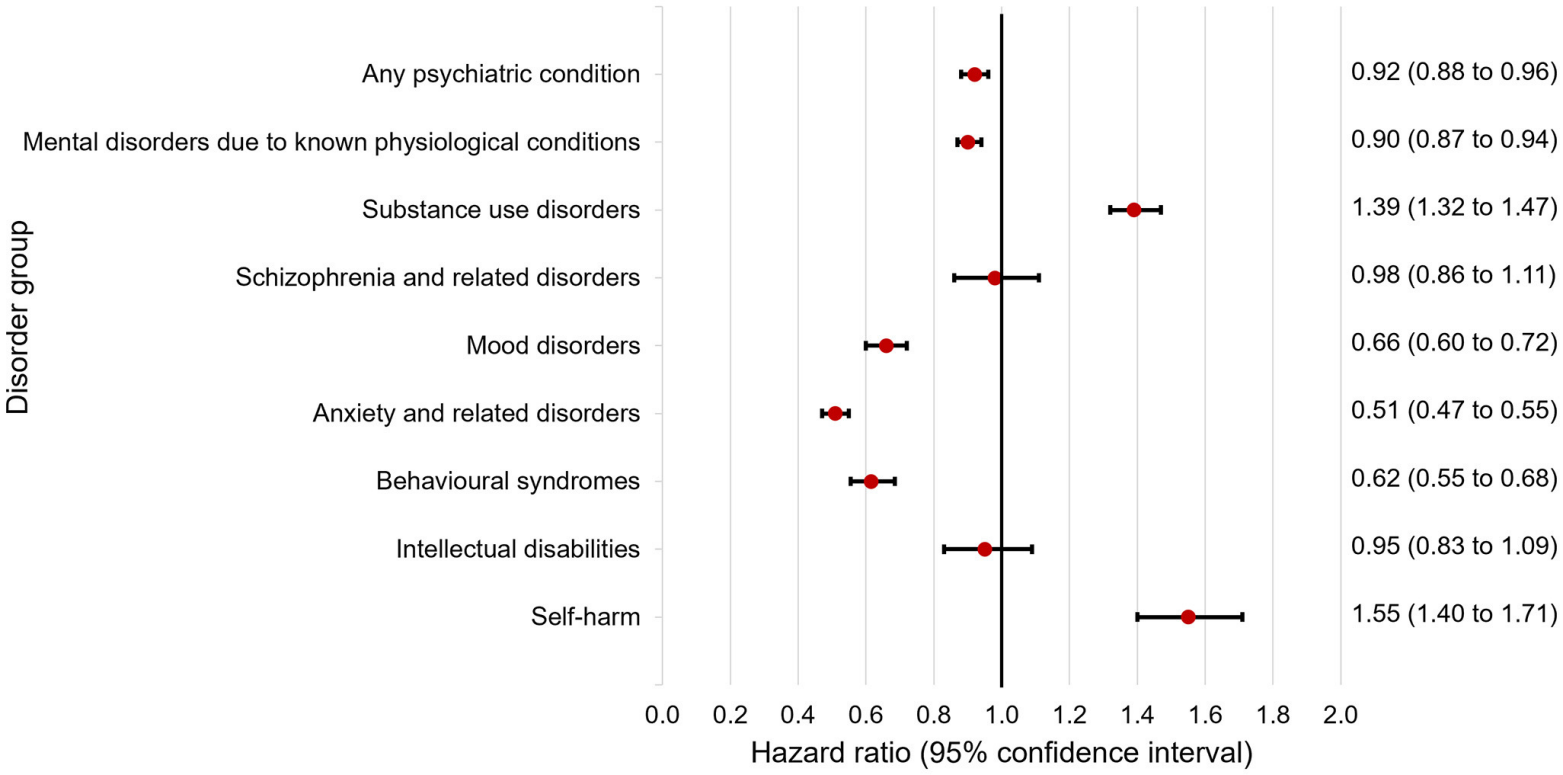
Hazard ratios for death by specific disorder groups after adjustment for age, sex, hospitalizations, days hospitalized, and the epilepsy-specific comorbidity index.

After including hospitalizations, hospital days and the epilepsy-specific comorbidity index in the Cox regression model, the diagnosis of any psychiatric comorbidity was not associated with elevated mortality (Figure 1). A respective result persisted after additional adjustment for the time of entry in the study, epilepsy type, the number of different epilepsy diagnoses or after including only adults in the model (Supplementary Table 1). Having a diagnosis of focal (HR = 0.74 95% CI = 0.71 to 0.77), generalized (HR = 0.70 95% CI = 0.66 to 0.74), or other (HR = 0.92 95% CI = 0.86 to 0.98) epilepsy was associated with decreased, while being diagnosed with special epileptic syndromes (HR = 1.42 95% CI = 1.34 to 1.51) or unspecified epilepsy (HR = 1.17 95% CI = 1.11 to 1.23)—with increased mortality in proportional hazards models including the diagnosis of any psychiatric comorbidity as a covariate. Epileptic encephalopathy and mixed seizure types (HR = 0.93 95% CI = 0.82 to 1.05) were not related to mortality in such a model.

## Discussion

In the current study we explored the association between psychiatric comorbidities and mortality in epilepsy by using a database of electronic health records from a population of 2.8 million. The rate of mortality in the current sample was high: this finding is likely associated with recent growth in mortality among PWE, which has been documented in Western countries and is thought to be determined by the overall growing burden of neurological diseases and lethal comorbidities in aging populations (17, 18). For instance, an increase from 16.9 deaths per 1,000 PWE in 2005 to 36.6 in 2013 has been recorded in the UK based on data from the Clinical Practice Research Datalink (17). Despite a high absolute mortality rate, the standardized mortality ratio of PWE in Lithuania is expected to be around three—a value similar to those reported in other high-income countries (14, 19). The non-exclusion of PWE with severe comorbidities that are not directly related to epilepsy (e.g., cardiovascular disease, dementia, cancer) may be another factor contributing to the high overall mortality rate (17, 18). While the study period included the first months of the COVID-19 pandemic, this event was most likely unrelated to the high mortality among PWE as COVID-19-associated infection and deaths rates remained low in Lithuania in the first half 2020 because of a strict nationwide lockdown (20).

The prevalence of psychiatric comorbidities among PWE was comparable to results by Fazel et al. who employed similar ICD-based diagnostic criteria, but the frequency of individual disorder groups was lower than in the study by Tao et al. who relied on data from neuropsychiatric interviews (9, 10). A lower prevalence of psychiatric comorbidities in studies relying on electronic healthcare databases is likely determined by widespread underdiagnosis of some psychiatric conditions (21, 22). Initially, we could confirm that the diagnosis of a psychiatric disorder is related to elevated age and sex-adjusted mortality among PWE, similarly as in the previous trials. However, the association was not present if the epilepsy-specific comorbidity index and hospitalization-related data were included in the model. We believe this can be explained by the methodological features of the study. For instance, around half of all PWE with a psychiatric comorbidity were diagnosed with a “mental disorder due to known physiological conditions.” This ICD category includes disorders, such as dementias or delirium, whose etiology is traceable to somatic illnesses. While such causality-based grouping has been criticized for creating “an intrinsic contradiction” within a descriptive classification of diseases, it also helps to distinguish between independent psychiatric comorbidities and somatic disease-associated psychiatric disturbances (23, 24). Given the high prevalence of the latter, the diagnosis of any psychiatric disorder within our PWE sample also indirectly reflected the burden of somatic illnesses. After adjusting for somatic illnesses and hospitalisations, on the other hand, there were only two groups of disorders independently linked to increased all-cause mortality: substance use disorders and self-harm. The misuse of psychoactive substances such as alcohol potentially has multiple effects on mortality among PWE—it highly increases the risk of death by external causes (e.g., trauma, suicide or homicide) and can underlie worse epilepsy care (e.g., infrequent outpatient visits, non-adherence to therapy) or cause other somatic and psychiatric conditions that are associated with poor outcomes (5, 10). Self-harm can be detected with acceptable sensitivity through administrative data and is associated with high and persistent risk of suicide (25, 26).

Mood, anxiety and behavioral disorders were hypothesized either to have no association with all-cause mortality or to increase it. An inverse relationship between these psychiatric comorbidities and odds of dying was unexpected as it is known that psychiatric disorders increase the risk of death from external causes (10, 27). While recent discussions based on mixed study samples suggest that the link between depression and all-cause mortality is not robustly proven, there is also no evidence for an inverse association (28). Some studies in epilepsy further indicated that there is no relationship between depression and all-cause mortality (9, 11). It is noteworthy that during the development of the epilepsy-specific index, the prevalence of depression alone was 28.2% [St. Germaine-Smith et al. (11)] and it attained 37% in the study by Tao et al. (9). A recent study by Wojewodka et al. report findings from a retrospective cohort, in which a third of PWE had depression that was also associated with 1.67 times increased odds of mortality. High depression rates correspond to those expected based on estimates from clinical interviews or psychometric scales (6). In contrast to such data, PWE with any mood disorder comprised only 7.7% of our sample. Thus, our result likely reflects the low sensitivity of ICD-based coding used in the study (21, 29). As most occurrences of mood or anxiety disorders remained undiagnosed within the sample, a documented diagnosis of such disorders was probably indicative of appropriate medical care and treatment, which in turn might paradoxically increase the odds of survival. Future prospective cohort studies from healthcare systems capable to better identify mood disorders could elucidate what is true relationship between these comorbidities and mortality in epilepsy.

Several limitations of our study should be cited. First, we used data from an administrative insurance database that was not designed for outcome analysis. Despite adequate specificity, the sensitivity for psychiatric disorders may be low if such electronic health records are used (21). Second, our analysis was limited to the sample of PWE alone and their survival was not compared with individuals without epilepsy. Similarly, the characteristics and mortality of PWE with psychiatric comorbidities could not be compared with a healthy control group. Third, the socioeconomic status and causes of death among PWE were unknown in our study, thus limiting the investigation to general survival analyses. Moreover, we used a case definition of epilepsy that did not separately consider the use of antiseizure drugs or require several consecutive records of the same diagnosis of epilepsy. This may result in a lower specificity of the dataset for the ascertainment of epilepsy cases. Finally, we were also unable to investigate the impact of medication or the use of other treatment methods (e.g., psychotherapy) on mortality among PWE. Given these important limitations as well as the single-country design of our study, the results may not be generalizable to other regions or directly comparable with studies employing data from neuropsychological interviews to detect psychiatric comorbidities. Future studies using data from registries or conducted prospectively are required to either confirm or refute findings reported in this investigation.

## Conclusion

The results of the current study contradict previous reports suggesting that psychiatric comorbidities increase all-cause mortality among PWE. Our findings indicate that the diagnosis of any psychiatric comorbidity is not associated with elevated all-cause mortality if somatic comorbidities are considered. However, self-harm and substance use disorders were independently related to higher rates of death during the study period. Future prospective studies based on clinical interviews should further investigate whether psychiatric comorbidities in epilepsy increase the odds of death in epilepsy after adjusting for somatic disorders.

## Data availability statement

The original contributions presented in the study are included in the article/Supplementary material, further inquiries can be directed to the corresponding author/s.

## Ethics statement

The study was conducted according to the guidelines of the Declaration of Helsinki and approved by the Vilnius Regional Ethics Committee for Biomedical Research (protocol code: LNLP-1, date of approval: 31 March 2020). Written informed consent for participation was not required for this study in accordance with the national legislation and the institutional requirements.

## Author contributions

KP, EK, and RM: conceptualization, methodology, data curation, and writing (review and editing). KP: software, formal analysis, and visualization. KP and RM: writing (original draft). EK and RM: investigation, resources, supervision, and project administration. EK: funding acquisition. All authors contributed to the article and approved the submitted version.

## Funding

The Joint Action CHRODIS+ has received funding from the European Union in the framework of the Health Programme (2014–2020) and provided primary data for the study. The funder was not involved in the study design, collection, analysis, interpretation of data, the writing of this article, or the decision to submit it for publication.

## Conflict of interest

The authors declare that the research was conducted in the absence of any commercial or financial relationships that could be construed as a potential conflict of interest.

## Publisher's note

All claims expressed in this article are solely those of the authors and do not necessarily represent those of their affiliated organizations, or those of the publisher, the editors and the reviewers. Any product that may be evaluated in this article, or claim that may be made by its manufacturer, is not guaranteed or endorsed by the publisher.
